# Electrocardiographic Imaging for Atrial Fibrillation: A Perspective From Computer Models and Animal Experiments to Clinical Value

**DOI:** 10.3389/fphys.2021.653013

**Published:** 2021-04-30

**Authors:** João Salinet, Rubén Molero, Fernando S. Schlindwein, Joël Karel, Miguel Rodrigo, José Luis Rojo-Álvarez, Omer Berenfeld, Andreu M. Climent, Brian Zenger, Frederique Vanheusden, Jimena Gabriela Siles Paredes, Rob MacLeod, Felipe Atienza, María S. Guillem, Matthijs Cluitmans, Pietro Bonizzi

**Affiliations:** ^1^Biomedical Engineering, Centre for Engineering, Modelling and Applied Social Sciences (CECS), Federal University of ABC, São Bernardo do Campo, Brazil; ^2^ITACA Institute, Universitat Politècnica de València, València, Spain; ^3^School of Engineering, University of Leicester, United Kingdom and National Institute for Health Research, Leicester Cardiovascular Biomedical Research Centre, Glenfield Hospital, Leicester, United Kingdom; ^4^Department of Data Science and Knowledge Engineering, Maastricht University, Maastricht, Netherlands; ^5^Electronic Engineering Department, Universitat de València, València, Spain; ^6^Department of Signal Theory and Communications and Telematic Systems and Computation, University Rey Juan Carlos, Madrid, Spain; ^7^Center for Arrhythmia Research, University of Michigan, Ann Arbor, MI, United States; ^8^Biomedical Engineering Department, Scientific Computing and Imaging Institute (SCI), and Cardiovascular Research and Training Institute (CVRTI), The University of Utah, Salt Lake City, UT, United States; ^9^Department of Engineering, School of Science and Technology, Nottingham Trent University, Nottingham, United Kingdom; ^10^Cardiology Department, Hospital General Universitario Gregorio Marañón, Instituto de Investigación Sanitaria Gregorio Marañón, and Facultad de Medicina, Universidad Complutense de Madrid, Madrid, Spain; ^11^Department of Cardiology, Cardiovascular Research Institute Maastricht, Maastricht University, Maastricht, Netherlands

**Keywords:** electrocardiographic imaging, cardiac arrhythmias, atrial fibrillation, inverse solution, AF characterization, catheter ablation, treatment guidance

## Abstract

Electrocardiographic imaging (ECGI) is a technique to reconstruct non-invasively the electrical activity on the heart surface from body-surface potential recordings and geometric information of the torso and the heart. ECGI has shown scientific and clinical value when used to characterize and treat both atrial and ventricular arrhythmias. Regarding atrial fibrillation (AF), the characterization of the electrical propagation and the underlying substrate favoring AF is inherently more challenging than for ventricular arrhythmias, due to the progressive and heterogeneous nature of the disease and its manifestation, the small volume and wall thickness of the atria, and the relatively large role of microstructural abnormalities in AF. At the same time, ECGI has the advantage over other mapping technologies of allowing a global characterization of atrial electrical activity at every atrial beat and non-invasively. However, since ECGI is time-consuming and costly and the use of electrical mapping to guide AF ablation is still not fully established, the clinical value of ECGI for AF is still under assessment. Nonetheless, AF is known to be the manifestation of a complex interaction between electrical and structural abnormalities and therefore, true electro-anatomical-structural imaging may elucidate important key factors of AF development, progression, and treatment. Therefore, it is paramount to identify which clinical questions could be successfully addressed by ECGI when it comes to AF characterization and treatment, and which questions may be beyond its technical limitations. In this manuscript we review the questions that researchers have tried to address on the use of ECGI for AF characterization and treatment guidance (for example, localization of AF triggers and sustaining mechanisms), and we discuss the technological requirements and validation. We address experimental and clinical results, limitations, and future challenges for fruitful application of ECGI for AF understanding and management. We pay attention to existing techniques and clinical application, to computer models and (animal or human) experiments, to challenges of methodological and clinical validation. The overall objective of the study is to provide a consensus on valuable directions that ECGI research may take to provide future improvements in AF characterization and treatment guidance.

## Introduction

Atrial fibrillation (AF) is a chronic condition with an overall prevalence rate of 2.9% (Benjamin et al., 2019), and is associated with increased morbidity and mortality. The electro-structural remodeling undergone by the atrial myocardium in patients affected by AF causes the disease to become increasingly sustained, and more challenging to treat (Nattel et al., 2008). The multi-scale mechanisms (molecular, cellular, neurohumoral, and hemodynamic) and the wide range of comorbidities that contribute to promote this remodeling are complex (Heijman et al., 2020). Additionally, at an early stage (paroxysmal AF) the frequency, duration, and burden of AF shows a large inter-individual variability (Wineinger et al., 2019). All in all, the progressive nature, complexity, and inter-patient variability of the disease make effective treatment of AF challenging (particularly in persistent AF patients), and require both gaining detailed information about the mechanisms underlying AF and its natural course, and a multidisciplinary approach to its management (Hindricks et al., 2020).

Therefore, identification and quantification of mechanisms of generation and maintenance of AF may help provide a more adequate AF stratification and guide AF therapy (Kirchhof et al., 2012). These mechanisms and the consequent remodeling of the atrial myocardium influence the propagation of the electrical activity in the atria both in sinus rhythm and during AF. This suggests that body surface potentials, for example captured by the clinical electrocardiogram (ECG), may retain information about the underlying electro-structural substrate of AF, and could be exploited for its identification and characterization (Guillem et al., 2013). In this respect, Marques et al. showed that analyses in the frequency and phase domain of Body Surface Potential Mapping (BSPM) recordings allowed the non-invasive characterization of rotors and their localization in the atria, and helped distinguish rotors from other mechanism (like ectopic foci or macro re-entrant circuits; Marques et al., 2020a). However, the electrical activity recorded on the body-surface is a smoothed and attenuated combination of all electrical activity at the level of the heart surface, which may limit the possibility to accurately identify and characterize AF mechanisms by only using the ECG (van Oosterom, 2012).

**FIGURE 1:**
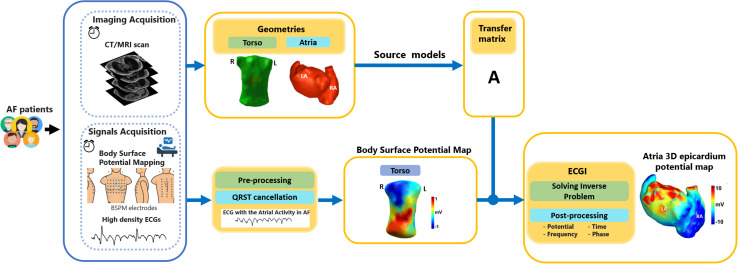
Steps required for the estimation of the electrical potentials on the atrial epicardial surface by means of Electrocardiographic imaging (ECGI). Body-surface potentials are measured by means of a dense array of electrodes, and the torso and heart geometries are acquired through magnetic resonance imaging (MRI) or computerized tomography (CT). The atrial surface potentials are then reconstructed by adopting a specific source model (surface-potential model in the figure). Figure modified from Romero (2019).

Electrocardiographic imaging (ECGI) may help obtaining a more detailed perspective, directly at the level of the myocardium. ECGI allows non-invasively estimating the electrical activity on the heart surface from a dense array of body-surface ECG recordings and a patient-specific heart-torso geometry (Rudy, 2013). ECGI has been widely used for the reconstruction of the activation and recovery sequence of the heart, the origin of premature beats or tachycardia, the anchors of re-entrant arrhythmias and other electrophysiological quantities of interest, both for improving diagnosis and for guiding therapy (Cluitmans et al., 2018). Figure 1 illustrates the standard steps of an ECGI procedure (with focus on the reconstruction of the heart potentials on the atrial epicardial surface).

ECGI determines the cardiac electrical sources for a given body-surface potential distribution, which is known as the inverse problem in electrocardiography (MacLeod and Buist, 2010). This problem is “ill-posed,” meaning that it does not have a unique solution and that the solution is highly sensitive to small changes in the input (because of measurement noise and inaccuracies in the estimate of the heart-torso geometry, see Supplementary Material; Colli-Franzone et al., 1985; Kabanikhin, 2008; Pullan et al., 2011; Rudy, 2013). A common approach to overcome these issues is to use regularization. Consequently, this makes ECGI strongly dependent on implementation choices, such as the cardiac source model and the method chosen for regularization.

In addition to these challenges, which apply to ECGI in general, others specific to targeting the atria during AF must be included. First, all of the existing different hypothesized mechanisms of generation and maintenance of AF (repeated rapid focal activity, Lee et al., 2015; rotors, Jalife, 2010; disrupted conduction of multiple stable wavelets that become fragmented, Allessie et al., 1996, 2010) regard atrial activity propagation patterns to be highly irregular and consequently more difficult to reconstruct than regular rhythms. Second, there are unique challenges when imaging the atria. Atria have very thin walls (ca. 4 mm), which are difficult to detect without submillimetre image resolution (McGann et al., 2014), compared to the ventricles (ca. 10 mm thickness). Additionally, atrial anatomy is more complex, with several small but important anatomical structures, such as the pulmonary veins and atrial appendages, which make it difficult to create accurate atrial models without high-resolution three-dimensional scans.

Therefore, in order to study the benefits of using ECGI in AF characterization and treatment, and help contribute to establish its clinical value in AF management, the aims of this paper are:

1. To review the current status of the use of ECGI in characterization, diagnosis, and treatment of AF.
2. To point out the current advantages and limitations of ECGI in investigating and characterizing AF, and suggest the range of clinical AF-related applications for which ECGI shows to be (potentially) useful.
3. To provide a consensus on what are the directions ECGI should develop in the future to answer the still open needs regarding the diagnosis and treatment of AF.

This manuscript is a joint effort of the atrial arrhythmias working group of the Consortium for ECGI (CEI)^1^, an international working group with the objective of facilitating collaboration across the research community in ECGI and creating standards for comparisons and reproducibility (Coll-Font et al., 2016).

In the next sections, we will highlight the mechanisms of AF (section “AF mechanisms”), methodological considerations for the use of ECGI in AF (section “ECGI in AF: Methodological Considerations”), previous validation efforts for ECGI in AF (section “ECGI Validation in AF: Outcomes From Mathematical Modeling, Animal Models, and Patients”), and its clinical application (section “Clinical Application: From Clinical Need to Workflow Integration”).

## AF Mechanisms

**FIGURE 2:**
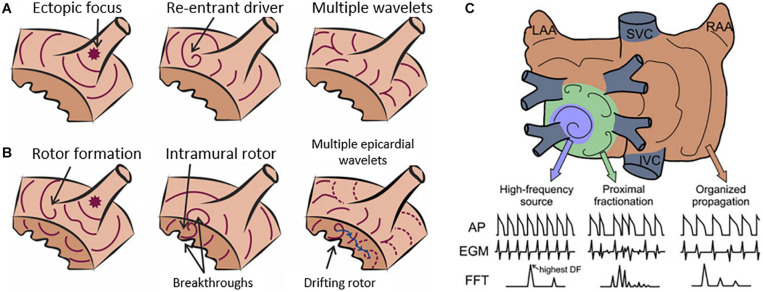
Current hypotheses for atrial fibrillation (AF) maintenance. **(A)** Diagram of AF maintenance near a pulmonary vein driven by ectopic focus (left), re-entrant driver (middle), or multiple wavelets (right). **(B)** Re-entrant drivers, or rotors, can be initiated by wave breaks near an ectopic focus (left) and underlie endocardial or epicardial breakthroughs (middle). A drifting rotor (right) can be the driver of multiple and apparently disorganized atrial wavelets. **(C)** Re-entrant drivers in general, present some spatiotemporal periodicity, and thus electrograms (EGMs) are regular. Spectral analysis identifies a dominant peak that matches the activation frequency of the re-entrant driver, which is the fastest across the atria. At the periphery of the re-entrant driver, propagation is disrupted as some activations are blocked. The variations in activation times and directions at the boundaries of the re-entrant driver result in EGMs with variable morphology and fractionation, with multiple peaks in the power spectrum. At more distal sites, the activation rate is reduced leading to fewer wave breaks and a more regular activity. Modified from Guillem et al. (2016).

AF is characterized by a fast and irregular atrial activity, with between 300 and 600 activations per minute. Unlike many other cardiac arrhythmias (e.g., ventricular ectopic beats or atrial flutter), AF may present irregular rhythm without a clear repetitive pattern of activation for some patients. During the last decades, several mechanisms of initiation and maintenance of AF have been proposed, from specific regions that drive the arrhythmia by means of ectopic sources or quasi-stable re-entrant drivers (functional re-entrant rotor mechanisms or structural micro-anatomic re-entries), to disrupted conduction of multiple stable wavelets that become fragmented (Figure 2; from Allessie et al., 1996, 2010; Jalife, 2010; Lee et al., 2015; Guillem et al., 2016).

It is also possible that multiple AF driving mechanisms may occur in the same patient. Recently, two additional mechanisms were proposed: endo-epicardial dissociation, suggesting that fibrillation could be sustained by an electrical dissociation of the intramural layers of the myocardium (Allessie and de Groot, 2014; Zaman et al., 2017), and re-entrant driver instability due to fibrosis, suggesting that re-entrant drivers are mainly present in the periphery of areas of the myocardium identified with a high degree of fibrosis (Hansen et al., 2015; Stanley, 2017).

Due to these complex electrical activations, invasive cardiac mapping technologies routinely used do not allow to fully capture the electrical patterns during AF. Electro-anatomical mapping systems are based on a sequential mapping of the cardiac chambers and require the presence of repetitive patterns to synchronize electrical recordings obtained at different locations at different time frames, and this requirement is not met during AF. On the other hand, basket catheters do allow for simultaneous recordings at multiple locations of the atria, but coverage of the atrial chambers is sometimes poor and leaves unmapped areas. ECGI, in contrast, offers a panoramic and simultaneous view of both atria with the potential to overcome these limitations of invasive mapping systems at the expense of a presumably lower spatial resolution, meant as the precision of the mapping obtained with ECGi (and depending on the number of surface electrodes employed). This can be estimated for instance in terms of localization error of site(s) of origin of an arrhythmia. Recent studies on animals and humans have shown median errors in the ventricles of about 13 mm in animals (interquartile ranges of about 7–22 mm, with at least 184 surface electrodes; Cluitmans et al., 2017; Bear et al., 2018), and of about 11 mm in humans (interquartile ranges of about 4–21 mm, with at least 130 surface electrodes; Graham et al., 2019; Parreira et al., 2020).

## ECGI in AF: Methodological Considerations

The physics of ECGI is governed by the Maxwell electromagnetic wave equations (see mathematical description of ECGI in Supplementary Material), where the electric field generated by the excitation of the heart is propagated to the surface of the body through a volume with passive electrical conduction (Rudy and Burnes, 1999; Rudy, 2013). In ECGI, heart signals are reconstructed by means of the Laplace equation within the torso volume conductor, using both the electrical potentials from the torso and the geometric relationship between the surfaces of the heart and torso (Rudy and Burnes, 1999; Rudy, 2013). Typically, the “cardiac source” is represented by extracellular potentials (for example unipolar electrograms at the epicardium), by transmembrane voltages, or by simplified activation/recovery models (Cluitmans et al., 2018). For application in AF, typically only the first cardiac source is used, and we will not discuss the other source models.

A sufficient number of electrodes has to be placed on the torso of the patient in order to characterize appropriately the electrical potentials on the torso surface. A seminal paper by Lux et al. (1978) quantified the number of leads required for a full reconstruction of cardiac surface potentials to be 30. The same approach was applied by Guillem et al. (2009a), specifically for recordings during AF with a very similar conclusion, with 34 leads as the limit for the signals to incorporate independent information above the noise level. Later, Marques et al. (2020b) showed that it is possible to even characterize atrial rotors on the body surface with as few as 32 leads. A more recent study quantified the number of leads required for accurate localization of the origin of atrial or ectopic beats and concluded that 74 leads are necessary (Parreira et al., 2020). Methodological differences can explain the difference in these results, since in the latter study leads were removed in bands and a homogeneous replacement of the re-positioned leads was not performed.

The next step in the ECGI procedure is the discretization of the surfaces of the heart and torso into triangular elements, allowing the relationship between torso and heart potentials to be represented with a linear model (a matrix equation). For atrial applications, this results in a “transfer matrix” describing the electrical relationship between electrical signals at the atrial surfaces and electrical signals at the body surface (see sections “Imaging Modalities for Personalized Anatomical Geometry Creation for Atrial ECGI Applications,” “Defining the Personalised Torso-Heart Electromagnetic Relationship,” and Supplementary Material). Discretization can be achieved through the Finite Difference, Finite Element or Boundary Element Methods (FDM, FEM, and BEM, respectively), resulting in a linear relationship of the transfer matrix, which contains the geometric information and electrophysiological properties of the volume conductor that relates the two surfaces (atrium and torso—Rudy and Burnes, 1999; Rudy, 2013).

Due to the aforementioned stability issue of ill-posedness in the inverse problem of electrocardiography, small real-world perturbations in the measurement of the torso signals (i.e., noise), or in the estimates of the torso and heart geometries and the electrode locations, can result in large errors in the calculation of the heart’s potentials (Colli-Franzone et al., 1985; Rudy and Burnes, 1999; Kabanikhin, 2008; Pullan et al., 2011; Rudy, 2013). Consequently, regularization methods are employed to force a unique and stable solution, with Tikhonov regularization (Tikhonov and Arsenin, 1977) being the most used method for this purpose (Figuera et al., 2016). Tikhonov regularization imposes constraints on the estimated heart potentials by delimiting their amplitudes or derivatives in space, time, or both (Rudy, 2013). Prior information can be added to the regularization to further constrain the solution space (Kabanikhin, 2008; see section “Regularization Methods” and Supplementary Material).

Additionally, the reconstructed atrial potentials need processing before interpretation. Particularly during AF, advanced analysis techniques are required to extract meaningful information from the noisy electrograms (see section “Post-processing for Reconstruction Interpretation”). The main implications and limitations of methodological choices in ECGI will be discussed at the end of section “Limitations and Challenges.”

### Imaging Modalities for Personalized Anatomical Geometry Creation for Atrial ECGI Applications

Medical imaging is a key component of ECGI systems to develop the personalized anatomical models of cardiac source, the atria and ventricles, and torso volume conductor. This requires including three-dimensional image acquisition, high speed, low cost, and common clinical availability (Cluitmans et al., 2018), together with accounting for atrial specific imaging-related challenges with respect to resolution.

The most common medical imaging modalities used in ECGI applications are CT and MRI (Ramanathan et al., 2004; Dubois et al., 2015). CT is the technique most ubiquitously used in ECGI applications because it is fast, low cost, globally available at most healthcare centers and provides high resolution (typical voxel size 0.5 × 0.5 × 0.5 mm). However, CT exposes patients to ionizing radiation and does not discern soft tissue types well. MRI requires no ionizing radiation exposure and has better tissue characterization capabilities but has significant barriers such as high cost (2–10 times a CT scan), need for specialized magnetic compatible equipment, overall lower resolution (voxel size 0.625 × 0.625 × 1.5 mm) and significantly longer scan times with limited hospital availability (McGann et al., 2008, 2014). If only personalized anatomy of the atria and the torso volume are required, CT is currently the preferred choice. If information is needed on tissue characteristics (e.g., fibrosis in the atrial wall), contrast-enhanced MRI may be preferred.

Unfortunately, including MRI or CT scanning in all patients is one of the main limitations for the extensive clinical application of ECGI. Recently, Rodrigo et al. (2018) explored the ability of a new metric based on the inverse reconstruction quality for the location and orientation of the atrial surface inside the torso. This approach has also been proposed to solve spatial inaccuracies provoked by cardiac motion or respiration, as well as to use ECGI on torso and atrial anatomies from different medical image systems (Gisbert et al., 2020).

### Defining the Personalised Torso-Heart Electromagnetic Relationship

After obtaining patient-specific imaging data, defining the electrical and conductivity relationship between the surfaces of relevance requires identifying the relevant tissues and anatomical structures. The simplest application entails defining the cardiac source, in this case the atria, the volume conductor boundary, in this case the torso surface, and the locations of the torso surface electrodes. More detailed models can include structures such as the ventricles, bone, lung, muscle, fat, or blood (Bear et al., 2015). These models can be generated by segmenting or contouring tissue identified on the medical image by using manual, semi-automated, or fully automated methods (Tate et al., 2018). Most segmentation methods examine the relative image intensity values and select pixels or voxels which correspond to relevant anatomical structures. Manual segmentation requires the user to identify voxels that belong to specific anatomical structures. Semi-automated methods can use image intensity, relative location, and spatial filtering combined to threshold and intuitively identify various structures with limited manual user “clean up” (Tobon-Gomez et al., 2015). Other, fully automated machine learning or atlas-based approaches have been developed and implemented. However, their usability in an ECGI setting has not been fully explored (Xiong et al., 2021). Specific segmentation software include many open-source and commercial tools such as Seg3d, Slicer, ITK-SNAP, ImageJ, and others.

There are several methods to subsequently create computational meshes from the segmented structures including common approaches such as Delaunay triangulation, lattice cleaving, and hexahedral meshing directly from imaging nodes (Ruppert, 1995; Shepherd and Johnson, 2008; Bronson et al., 2013). Delaunay triangulation assumes a point cloud of nodes that represent anatomical boundaries and creates triangular surface elements connecting the nodes that minimize each surface element aspect ratio (Ruppert, 1995). This method is suitable for ECGI applications because it can accurately conform to any anatomical boundary. Lattice cleaving creates similar quality meshes with significantly less computational overhead (Bronson et al., 2013). Hexahedral meshing assumes a rectangular element and is limited to structures with regular shape, hence it is not commonly used in ECGI applications (Shepherd and Johnson, 2008). Less common approaches, such as the method of fundamental solutions, can compute the cardiac sources without defining mesh connectivity (which may sometimes be challenging) and only use the boundary node locations (Wang and Rudy, 2006). There are many different software packages to create usable computational meshes, which implement a wide variety of different meshing approaches. Some popular meshing packages are cGAL, Tetgen, and Cleaver.

Ultimately, having computational meshes for the required torso and heart structures subsequently allows to calculate the electrostatic relationship between these surfaces, yielding a linear relationship between heart and torso potentials captured by the personalized transfer matrix (Barr and Spach, 1978).

### Regularization Methods

To deal with its sensitivity to noise, ECGI requires regularization to restrict the reconstructions to physiologically realistic solutions. Most studies apply the zero-order Tikhonov regularization method (see mathematical-based description of the method in the Supplementary Material), as Tikhonov-based regularization methods perform as well as more complex techniques in realistic fibrillatory conditions (Figuera et al., 2016). The Tikhonov regularized solution is obtained by minimizing an appropriate objective function (Pullan et al., 2011) to “regularize” (constrain) the inverse solution. A regularization parameter determines to what extent the final inverse solution depends on the Tikhonov regularization. A higher value of this parameter leads to a smoother solution, i.e., reduces noise more, but it can also remove localized activation patterns (an over-smoothed solution) (MacLeod and Buist, 2010). Within the Tikhonov regularization, one can choose between zero order regularization (based on the idea that epicardial potentials should be reasonably small), or first- or second-order Tikhonov (based on the idea that such potentials should be changing smoothly over the heart surface). Zero-order Tikhonov regularization sets the Tikhonov matrix to be the identity matrix, which effectively limits the total magnitude of the solution. First-order Tikhonov regularization sets the Tikhonov matrix to a discrete approximation of the surface gradient operator that limits the slope of the solution, and the second-order Tikhonov regularization sets the Tikhonov matrix to a discrete approximation of the Laplacian surface operator, to restrict the rate of change in the slope.

Other regularization methods that have been implemented for smoothing the inverse solution include Generalized Minimal Residual (GMRes), singular value decomposition (SVD), total variation (TV), Bayesian Maximum a Posteriori Estimation (Bayes) and MUltiple Signal Classification (MUSIC/Greensite) algorithms (Ramanathan et al., 2003; Ghosh and Rudy, 2009; Onal and Serinagaoglu, 2009; Figuera et al., 2016; Pereyra, 2017) (see methods description in the Supplementary Material). Briefly, the GMRes method is an iterative numerical method that does not require the imposition of constraints on the solution (Calvetti et al., 2000; Ramanathan et al., 2003). SVD algorithms used most frequently are truncated (TSVD) and damped (DSVD) SVD. In TSVD, the transfer matrix is truncated such that all its singular value components that represent noise are removed. As components representing noise usually have small singular values, the truncation is implemented by maintaining a set of k components with the highest singular values (Hansen, 2010). The value of k needs to be set *a priori* to obtain a solution. DSVD is a less “brute force” application compared to TSVD, as it allows a filtering of singular value components rather than forcing to make an inclusion/exclusion decision on the components to include in the final solution (Figuera et al., 2016). The Bayesian approach is based on *a priori* knowledge of the spatial covariance matrix and mean of the epicardial potentials, setting this mean to zero (Figuera et al., 2016). This approach only accounts for spatial correlation of the potentials. A temporal correlation can be included based on the isotropy assumption described by Greensite (2003).

In Figuera et al. (2016), 14 different regularization methods were compared in a simulation study, aimed at assessing the performance of regularization methods for reconstruction of atrial potentials during AF. The computational model simulated normal sinus rhythm as well as one simple and one complex AF conduction pattern, assuming perfect knowledge of the transfer matrix, and testing for different signal-to-noise ratios (SNRs). It was found that for the reconstruction of epicardial potentials, Bayes markedly outperforms the other methods, and zero-order Tikhonov with the instantaneous choice of the regularization parameter was the second best. A similar result held for phase maps and singularity point detection, though the best choice of the precise value of the Tikhonov regularization parameter was less pronounced. In such a simulation study, complete knowledge of the epicardial potentials was available, which gave the Bayes approach an extra competitive edge compared to real applications in which additional measurements must be performed to estimate those (Gribonval, 2011; Calvetti and Somersalo, 2018). In addition, results for different values of SNR showed that no algorithm was significantly more robust with regard to changes in noise level. Overall, the results of this study suggested that zero-order Tikhonov seems to be insensitive to moderate changes in regularization parameters, and offers a simple and pragmatic approach, although studies using real data are needed to corroborate these findings.

### Post-processing for Reconstruction Interpretation

After reconstruction, atrial signals can be used to create activation maps, localize rotors, or find other clinically relevant metrics. Up to quite recently, characterization of AF mechanisms (and of the areas responsible for its maintenance) in humans was performed exclusively by invasive mapping systems, consisting of introducing intracavitary catheters with multiple electrodes into the heart to map the electrical activity of the endocardium, and projecting electrophysiological characteristics on 3D maps of the atria. ECGI provides a non-invasive, instant-acquisition alternative to that approach. The main maps are isopotential, isochrone, frequency, and phase maps.

Using classical isochronal maps to analyze the temporal evolution of the irregular activation patterns would require generating an activation map for each of the 300–600 atrial activations that happen per minute. Despite this requiring a substantial effort, the patterns of AF mechanisms like ectopic activity or rotors observed through these maps have intrinsic characteristics that allow accurate characterization. Briefly, the ectopic activity is observed in isochrone maps as wavefronts that emerge from a focal origin and radially propagate to other areas with a uniform or anisotropic pattern. Rotor patterns in isochrone and phase maps present a rotational activity in which myocardial cells around a (non-excitable) nucleus have a progression of the phases starting at activation until repolarization, at which point the cycle starts again (Umapathy et al., 2010; Navara et al., 2018). However, multiple and continuous intra-atrial re-entry is generally observed with complex patterns when compared to the ectopic and rotor mechanisms. The collision of the re-entry circuits generates unstable and short-lived propagation behavior on phase and isochronous maps, sometimes indicating spurious rotors and ectopic activity.

Despite these advantages of isochronal maps, the detection of atrial activation times is a complex procedure, even visually in intracardiac contact catheters. To overcome those limitations, several signal processing approaches have been developed. One of the most popular is the so-called phase map analysis (Gray et al., 1998). Phase analysis has been applied to describe the spatiotemporal progression of the activation during AF (Guillem et al., 2016). In phase maps, wavefronts propagation is identified by assessing the complete morphology of each signal, without the need of determining the exact moment of activation.

An alternative approach to identify AF foci or conduction paths is through the frequency spectrum. The highest frequency of activation tends to be the “driving frequency” because once a cardiac myocyte is activated it goes into a refractory period, so the remaining areas of the atria will be activated passively at the pace of the fastest activated area, so that “the slower follow the fastest.” This has led to some success when using invasive atrial electrograms (Sanders et al., 2005; Atienza et al., 2009, 2014; Salinet et al., 2013b, 2014, 2017; Li et al., 2017). The next logical step is to localize those sites of highest dominant frequency, which are expected to be associated with the locations of ectopic activity and rotors, using ECGI. At the same time, it is important to bear in mind that while stable rotors and ectopic activity may provide relevant AF targets (e.g., for ablation), functional collisions can generate high frequency values that do not represent the areas responsible for maintaining AF.

A relevant challenge in the interpretation of signals and maps during AF is that in many cases these maps are complex and uncertain, hindering the correct characterization and location of arrhythmogenic sources. In addition, the electrograms of the area under analysis can be contaminated with far-field potentials. Other factors, such as the type of technology used by the manufacturer of the electrical mapping system, the map used, the duration of the analyzed signal (complex rhythms tend to reveal different patterns when signals of longer duration are analyzed), and the number of electrodes (spatial resolution) have been shown to influence the fibrillatory patterns identified in clinical research with patients. Up to quite recently, ECGI only allowed the identification of rotors located in the epicardium, with no evidence of ectopic activity in the same group of patients (Haïssaguerre et al., 2014). However, this might be due, among others, to the fact that pre-processing strategies applied to the body surface signals, as well as the resolution of the inverse problem used, may not be customized for AF, thus limiting its application.

Although initially intracavitary ablation procedures focused on reducing the available atrial tissue to prevent the maintenance of multiple wavelets (by means of extensive procedures that replicate the MAZE surgical strategy; Swartz et al., 1994; Haïssaguerre et al., 1996), the success of pulmonary vein (PV) ablation in terminating the arrhythmia in a significant number of AF patients shifted the attention to the identification of driver regions (Jaïs et al., 2005). PV isolation is nowadays considered the standard for AF ablation and is the first line approach in recent guidelines (Hindricks et al., 2020). However, AF recurrence typically occurs in more than 40% of paroxysmal AF patients after PV ablation (Medi et al., 2011; Weerasooriya et al., 2011). Moreover, in non-paroxysmal AF patients, success rates of single PV isolation are discouraging, with recurrence values from 28 to 51% (Chao et al., 2012).

### Limitations and Challenges

#### Noise, Transmural Aspects, and AF Stability

Several sources of noise, distortion, and inaccuracy may affect ECGI for AF. First, the effects of SNR of the recorded body-surface potentials have been scrutinized in AF simulations for additive Gaussian noise (Figuera et al., 2016), although Laplacian distributed noise has been suggested as a more likely statistical prior for bioelectric cardiac signals (Mincholé et al., 2014). Moreover, cardiac signals are usually low-pass filtered during their pre-processing, to improve SNR. It has also been pointed out that the basic equations of the inverse problem could be much more sensitive to noise in the geometrical conductivity relationship (captured by the matrix) than to noise in the recorded signals (Caulier-Cisterna et al., 2018). To the best of our knowledge, this has not been evaluated in detailed simulations or in real data, but it would be consistent with the ill-posed nature of the inverse problem. Finally, other sources of errors seem to have a strong impact on the solution, such as model bias or incorrect regularization adaptation. For instance, some controversy arose recently when activation mapping obtained with commercial ECGI systems seemed to exhibit differences with catheter recordings (Cluitmans et al., 2019; Duchateau et al., 2019; Rudy, 2019). In several cases these differences could be due to the estimated potentials being strongly biased due to over-smoothing.

A different source of limitations for current estimation methods is the interference of far-field ventricular activity on atrial signals during AF. Ventricular artifacts are markedly present on unipolar configurations, including intracardiac and ECGI signals. A way to remove ventricular interference in intracardiac unipolar signals, which can be extended to non-invasive signals, was proposed in Salinet et al. (2013a), where the authors showed that an adaptive template, built on a sliding-window and updated for each beat, using surface-ECG for QRS-T detection was an effective technique to avoid distortion on the estimation of frequency domain parameters related with AF from ventricular sources. Several other methods for ventricular far-field removal on non-invasive signals have been also proposed among which those presented in the following studies (Bollmann et al., 2006; Langley et al., 2006; Lemay et al., 2007).

Two assumptions on AF signals aiming to yield information about targets for ablation are temporal stationarity (when calculation of the spectrum is involved) and spatial consistency and stability (when parameter maps are obtained). Few studies have aimed to explicitly show the stationarity or cyclostationarity of AF signals in these conditions (which theoretically would support spectral decompositions) either in electro-anatomical mapping systems or in optical mapping recordings (van Hunnik et al., 2018). Spatio-temporal autocorrelation estimations have been recently proposed to support ECGI-based analysis on sustained rhythms (Caulier-Cisterna et al., 2020), which could be extended to the spectral-spatial domain definition. Most of this work is based on pinpointing ventricular activity and pacing sites, but may be translated to AF pattern analysis (e.g., Zhou et al., 2016). However, there is also evidence that AF is a progressive disease, going from paroxysmal to persistent to permanent AF, and it should also be taken into account that in AF the electrical activation markers may not always be spatio-temporal stationary or stable (De Vos et al., 2010; De Groot et al., 2010). In this respect, great efforts have been made during the last two decades to develop tools for the identification of ablation target regions in each individual patient that could improve the efficacy of PV ablation. Differently from other arrhythmias with regular patterns, AF beat to beat variability makes the analysis of the electrical activity using classical parameters such as activation time less adequate. Identification of drivers by considering the origin of an isochronal map, or the identification of the shortest pathway of a stable re-entrant circuit, is limited in a situation in which re-entrant circuits and their isochronal maps change from beat to beat. Consequently, identification of those atrial regions that could be responsible for driving AF requires more sophisticated analysis.

Other electrophysiological sources of possible uncertainty could involve the consideration of the intramural or endocardial myocardium and of the septum in the source estimations, though few studies have tried to characterize them, and the importance of their influence remains unknown. However, in recent years, the endocardial-epicardial dissociation has been shown by using endo-epi phase mapping on short and prolonged AF, thus suggesting that, even in the atria, complex 3-dimensional intra-wall structures could be relevant for successful ablation (Verheule et al., 2014; Parameswaran et al., 2020). Current ECGI systems still do not possess the required resolution, but such measurements could play a significant role in this scenario.

#### Volume Conductor Effects

The technique by which the volume conductor is accounted for mathematically affects the inverse solution, and therefore the accuracy of ECGI in describing AF electrophysiology. Generally, when traveling from the cardiac surface to the torso, the volume conductor is considered a spatio-temporal filter, which blurs detailed local atrial activations (Stinstra and Peters, 1998; Ramanathan and Rudy, 2001a; Nowak et al., 2006; Vanheusden et al., 2019). The accuracy of the solution can be affected by whether and how tissues and organs are included in the volume conductor model, as well as their localization and electric properties in the model (Gabriel et al., 1996; Klepfer et al., 1997; Ramanathan and Rudy, 2001a,b; Cheng et al., 2003; van Dam and van Oosterom, 2005; Potyagaylo et al., 2016, 2019; Tang et al., 2018).

The largest errors in inverse solutions related to the volume conductor arise from geometric errors (Hoekema et al., 2001; Cheng et al., 2003; Yao et al., 2016). Errors related to electrical properties of thoracic tissues appear limited (Ramanathan and Rudy, 2001a,b; Cheng et al., 2003; Potyagaylo et al., 2016). Geometric errors lead to deviations in localization of activation and potential features, whereas the lack of consideration for inhomogeneities (or misrepresentation of their electrical properties) leads to a change in morphology of these features (Klepfer et al., 1997; Ramanathan and Rudy, 2001a,b; van Dam and van Oosterom, 2005). Activation-based methods appear more robust to geometric errors (Cheng et al., 2003). A study using the fastest route algorithm as initial estimate for activation time imaging found the algorithm robust against lead and geometric errors (Potyagaylo et al., 2016). Reducing the need for incorporation of inhomogeneities may lead to the development of inverse solutions that are free from the need for patient-specific imaging. Recent simulation studies have shown the potential of detecting atrial location based on a Pattern Search algorithm on the L-curve curvature from the Tikhonov regularization method (Rodrigo et al., 2018; Gisbert et al., 2020).

To accurately reconstruct atrial activations, one study suggested that lungs and cardiac blood cavities within the thorax need to be correctly localized and described with realistic electrical properties (van Dam and van Oosterom, 2005). It was shown that inaccurate estimations of conductivities especially affected P-wave morphology (van Dam and van Oosterom, 2005). However, a similar setup also appears sufficient for determining the effect of ablation as well as contribution of the right and left atrium to the activity seen on the body surface (Jacquemet, 2015). One study simulating atrial repolarization, which used a reaction-diffusion system to reduce errors, showed that a homogeneous volume conductor is sufficient for reconstructing a realistic transmembrane potential distribution (Tang et al., 2018).

Another source of error is cardiac motion and respiratory movement. As shown in Cluitmans and Volders (2017) ventricular motion is associated with reduced reconstruction accuracy since when using a constant transfer matrix implies that due to the cardiac motion, the potentials are reconstructed on the wrong point in the epicardium.

If ECGI is used to determine spectral and temporal distributions on the AF propagation patterns, further understanding is needed on how the inverse algorithm attempts to compensate for the smoothening imposed by the volume conductor on the body surface signals, and on how it can potentially induce noise on the reconstructed atrial signals (such an understanding, in turn, requires insight about the mathematical and computational aspects of the inverse algorithm, Hansen, 2010). This also includes better characterizing the effect of regularization on the inverse solution.

Overcoming the above highlighted limitations/challenges is of great importance to the field. One could consider adding atrial wall thickness as a potential field source if appropriate estimation methods using source volumes instead of surfaces are used (He and Wu, 2001). This would complicate current estimation algorithms since transmural anatomy should be accounted for. The effect of taking this into account is yet unclear and it would require further investigation. Through a Finite Element Method (FEM) volume conductor model, electrical properties of the atria myocardium can be considered, and it would be more accurate if muscle elements such as anisotropy are incorporated, since it affects the dissociation between epicardium and endocardium (Hansen et al., 2015) and their respective signal amplitudes (voltages).

With respect to the volume conductor effect, further studies should be carried out aiming at the investigation of the endo-epi dissociation (Gharaviri et al., 2017), where both epi and endocardial atrial electrical activity is acquired simultaneously and recorded alongside high-density mapping of body surface signals (Vanheusden et al., 2019). For certain cases, it would be of relevance to pace the atrial substrate to have prior knowledge of the source, as well as in protocols where ablation did return a patient to sinus rhythm (Ramanathan et al., 2006). Studies further evaluating the anisotropic behavior of the atrial muscle, for example through fitting of appropriate wavefront propagation function onto atrial activation maps as well as simulation studies (Roney et al., 2019) should be conducted to allow the effect of anisotropy to be incorporated in volume conductors.

Future methods to overcome the limitations in the sensitivity of inverse problem related to noise in the geometrical conductivity relationship should focus on at least the following two points: first, the reduction of uncertainty in the estimation of the transfer matrix, derived from medical images in a process that can accumulate errors in different stages (e.g., voxel discretization, segmentation errors, or BEM assumptions); and second, to explore other estimation methods that are less sensitive to matrix inversion, especially in case of large matrices as it is the case with ECGI.

Regarding the latter, machine learning algorithms have been used to tackle ill-posed problems in other fields, and they showed to be able to overcome more traditional regularization methods. For instance, kernel methods for classification or Gaussian Processes for regression (Rojo-Álvarez et al., 2018) showed to overcome Least Squares (with L1 and L2 regularization). Furthermore, recent progress in reconstructing head models have shown the potential to derive information about electrical conductivity of brain tissue using information from T1 and T2-weighted images from MRI (Rashed et al., 2020). These results allow us to envision that similar approaches might help in defining better the electrical properties of torso tissues in future systems. Similarly, neural networks may become of interest in spatial clustering and classification of ectopic atrial foci into atrial (ablation) regions from body surface maps (Ferrer-Albero et al., 2017).

## ECGI Validation in AF: Outcomes From Mathematical Modeling, Animal Models, and Patients

The mechanisms that initiate and maintain AF are still under debate, and consequently, optimal treatment targets remain undefined, as well as the optimal signal processing tools to identify such targets. In the literature, spectral components, instantaneous phase or global degree of organization (also known as AF substrate complexity) have been applied to characterize AF. All of them have their own advantages and drawbacks. In addition, the signal length seems to affect the analysis since complex rhythms tend to reveal different patterns when signals of longer duration are analyzed. This section provides a review of the studies evaluating and validating ECGI to measure those characteristics, together with the efforts done to determine how well the organization of AF propagation patterns can be estimated from the body surface. This in turn may help understand the potentials and limitations of ECGI in reconstructing this information at the level of the heart, and help understand the type of questions that can be reliably tackled by ECGI when it comes to diagnosis and treatment of AF.

### Validation of ECGI During AF in the Spectral Domain

**FIGURE 3:**
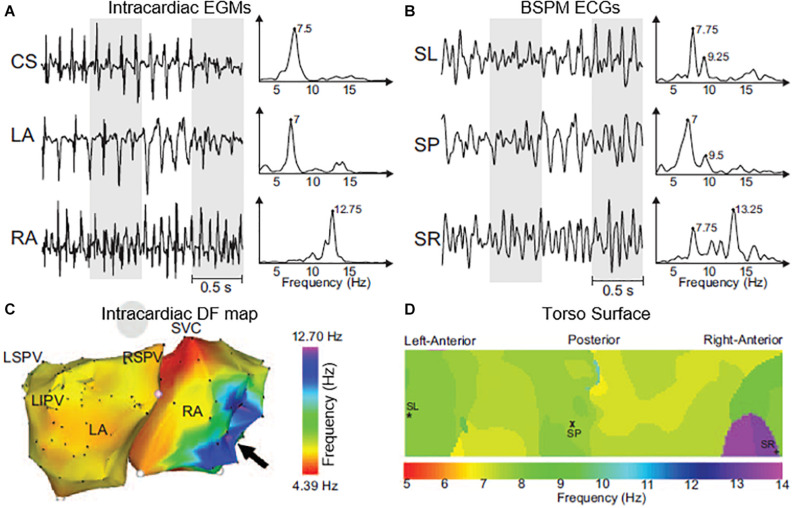
Intracardiac electrograms (EGMs) and body surface ECGs and their dominant frequency (DF) distribution in a sample patient with a right-to-left DF gradient. **(A)** EGMs recorded at different atrial sites and their corresponding power spectra. **(B)** Selected BSPM leads and their corresponding power spectra. **(C)** Intracardiac DF map. Black arrow points to the right atrial (RA) region with highest DF. **(D)** 2D DF map on the torso surface with superimposed locations of electrodes from **(B)**. CS indicates coronary sinus; LA, left atrium; LIPV, left inferior pulmonary vein; LSPV, left superior pulmonary vein; RSPV, right superior pulmonary vein; SL, surface left; SP, surface posterior; SR, surface right; and SVC, superior vena cava. Figure modified from Guillem et al. (2013).

**FIGURE 4:**
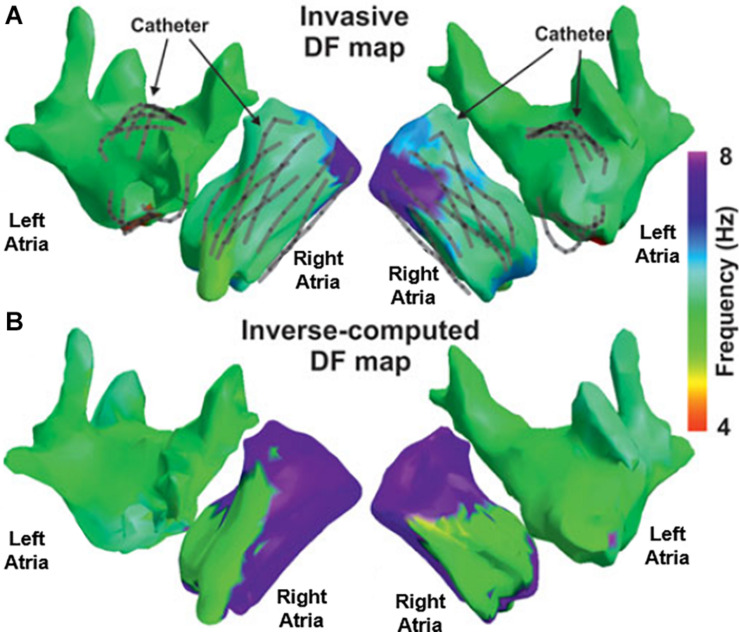
Validation of inverse computed DF maps. Electrocardiographic imaging (ECGI) inversely computed **(A)** and simultaneously recorded **(B)** DF maps obtained in a patient in which a multipolar catheter was sequentially placed in the right and left atria. Figure modified from Pedrón-Torrecilla et al. (2016).

A direct comparison between endocardial and BSPM recordings in AF patients showed the feasibility to non-invasively detect the highest dominant frequency (Guillem et al., 2013) in both atria (Figure 3). The areas of highest dominant frequency have been shown to correlate with the rotor core in both models and AF patients (Marques et al., 2020a,b). On the other hand, Vanheusden et al. found significant differences in highest dominant frequency values between intra-cardiac and torso signals, when evaluating the highest dominant frequency behavior in AF patients using simultaneously measured virtual atrial electrogram signals from both atria and BSPM (Vanheusden et al., 2019). As for ECGI specifically, both mathematical models and clinical recordings have been used to validate the accuracy of non-invasive mapping to identify dominant frequencies (Figure 4; Figuera et al., 2016; Pedrón-Torrecilla et al., 2016; Zhou et al., 2016; Rodrigo et al., 2017a; Cámara-Vázquez et al, 2021). Since computation of highest dominant frequency requires a time segment, it is obtained as the average over a certain period, and appears to be a more robust parameter in AF patients than instantaneous signal properties such as phase (Pedrón-Torrecilla et al., 2016). Persistent AF patients presented rotor drifting, but with spatial repetition of the highest dominant frequency sites. Paroxysmal patients showed left-to-right maximal frequency gradient, as opposed to the situation for persistent AF patients (Lazar et al., 2004; Atienza et al., 2006, 2009). Nevertheless, the combination of re-entrant analysis and highest dominant frequency regions has been suggested as an optimal approach to identify AF drivers from ECGI in a computational study by Rodrigo and colleagues (Rodrigo et al., 2017a). Non-invasive estimation of frequency of activation during AF showed to be more accurate with a wavelet-based approach rather than with Welch method in both models and AF patients (Marques et al., 2020b).

Although these spectral-domain metrics demonstrate reasonable correlation with invasive localization of AF drivers, their potential to manage AF therapy (e.g., guide ablation procedures) remains untested/unproven.

### Validation of ECGI During AF in the Time and Phase Domains

Due to the high activation rate, activation maps are typically not used during AF. Conversely, phase mapping has been applied to identify repetitive activation patterns (Guillem et al., 2016; Rodrigo et al., 2014). In 2D models of human atrial tissue and recorded in isolated sheep hearts, phase maps have been used to detect relatively stable re-entries and foci (Zlochiver et al., 2008). Additionally, as mentioned in the introduction, computational studies suggest that BSPM (without ECGI) may already provide a characterization of AF mechanisms, by allowing the non-invasive characterization of rotors and their location in the atria, and showing large areas of highest dominant frequency, with values close to the frequency of rotation of the atrium rotor (Marques et al., 2020a). Conversely, the mechanism of multiple and continuous intra-atrial re-entry is generally observed with unstable and short-lived patterns when compared to the ectopic and rotor mechanisms.

Importantly, phase mapping is only possible for simultaneously acquired signals, and is thus typically not available for invasive recordings, where data points are collected sequentially. Basket catheters do allow for simultaneous recordings at multiple locations of the atria. However, coverage of the atrial chambers is sometimes poor and leaves unmapped areas, which prevents from generating reliable phase mapping. Consequently, the lower resolution of ECGI compared to invasive recordings may be compensated by its ability to provide reliable phase mapping in the entire atrial surface. Nevertheless, the quality and number of phase singularities computed from ECGI may be dependent on using one or two volumes for the atria geometries, and it is likely that there exists a loss of information in the interatrial septum when two volumes are used. Some preliminary studies suggest that only one volume should be used (González-Ascaso et al., 2020), but this issue still remains unclear. Recent studies in AF patients correlated the rotors detected by invasive mapping with ECGI showing a high correlation in occurrence of 0.97 (Metzner et al., 2017). Additionally, detection of AF re-entrant sites in AF patients showed good correlation in non-invasive and invasive methods, and correlation between the number of simultaneous re-entrant regions (Rodrigo et al., 2020). Recently, a novel vector mapping approach called stochastic trajectory analysis of ranked signals (STAR) has been proposed as an alternative to the use of phase mapping for localizing AF drivers. This system can be used with sequential invasive recordings, and it could be successfully used to terminate AF during catheter ablation (Honarbakhsh et al., 2019, 2020).

### Validation of ECGI to Guide Ablation Therapy

**FIGURE 5:**
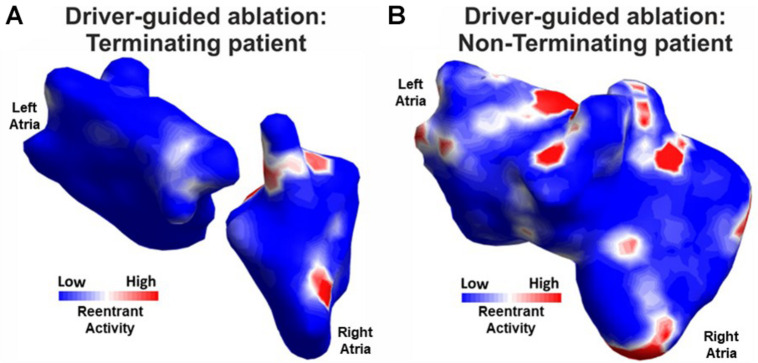
Re-entrant activity cluster maps from driver-guided ablations in terminating **(A)** and non-terminating patients **(B)** (modified from Rodrigo et al., 2020).

Several studies have reported ECGI as a valid tool to detect and localize re-entrant activity in the atria with the purpose of guiding clinical therapy. Identification of regions of AF generation and maintenance with ECGI in AF patients showed AF termination with ablation near those ECGI-detected sites (Figure 5; Cuculich et al., 2010; Haïssaguerre et al., 2013, 2014). This was also validated in a larger population of 103 persistent AF patients (Haïssaguerre et al., 2014), and proved to be helpful in shortening ablation times. In a multicenter study, good results were also reported using ECGI prior to ablation procedures for driver identification with favorable outcomes at 1 year, with 78% of the patients without AF recurrence (Knecht et al., 2017). In this study, ECGI was used in eight centers with no experience and similar results, showing the reproducibility of the technique and an easy adaptation in the clinical practice. Overall, the study showed that ECGI-ECVUE mapping system accurately identified focal and localized re-entry circuits, which after being ablated resulted in AF termination in 64% of 118 patients. 81% of the drivers were re-entrant, and 19% were focal (53% located in the left atrium, 27% in the right atrium and 20% in the anterior interatrial groove). Focal breakthroughs were mostly located on the PV ostia. Different types of phase singularity movements (figure-of-eight shaped re-entry; the phase front ends organized as a pair of rotors with opposite chiralities, rotating clockwise and counterclockwise) were frequently observed. Most of the majority of the identified rotors meandered, and their rotation was, in most cases, less than one full rotation. Importantly, ECGI was used in these studies to characterize AF mechanisms but no ground truth data were available to validate the outcomes, and when ECGI was used to guide therapy, no control group was used. Despite these limitations, these studies show the potential benefit of ECGI characterization of AF mechanisms.

More recently, ECGI was used for guiding ablation procedures and it was reported that it could be used for patient stratification since differences in driver locations were found non-invasively in patients with AF acute termination compared with patients with unsuccessful termination outcomes (Gao et al., 2019). In a later study, a moderate correlation between the number of simultaneous re-entrant regions with the outcome of AF ablation has also been reported (Figure 5; Rodrigo et al., 2020). Moreover, other recent studies showed the potential of preoperative non-invasive mapping for the characterization of AF in combination with other ablation procedures like Cox-Maze procedures, to personalize the intervention to the patient in combined cardiac surgeries (Ehrlich et al., 2019; Osorio-Jaramillo et al., 2020b).

By combining the information provided by ECGI with other structural information, such as fibrosis (obtained from late gadolinium MRI) has shown to be useful in identifying re-entrant activity around fibrotic areas (Roney et al., 2016; Cochet et al., 2018). In addition, modeling patient fibrosis and combining it with ECGI showed better results in AF characterization (Boyle et al., 2018), which is linked to the outcome of ablation strategies (Chelu et al., 2018), thus improving therapy guidance. Nevertheless, some authors reported ECGI-detected rotors are not directly associated with the location of fibrotic areas (Sohns et al., 2017), but suggested that personalized MRI-based atrial models in combination with non-invasive mapping could be used for guiding ablations in persistent AF patients (Sohns et al., 2018).

Notwithstanding the difficulties of finding a gold standard for ECGI validation in detecting AF mechanisms of generation and maintenance due to the low spatial resolution of reconstructed electrograms, ECGI technology has proven to be useful for identifying relevant AF drivers. Non-invasive mapping has been crucial in studies that explored new ablation therapies in addition to pulmonary vein isolation to restore sinus rhythm in AF patients (Haïssaguerre et al., 2014), by identifying AF drivers in other parts of the atria. Despite that, ECGI is still not typically used for ablation yet. This is mainly because it requires collection and analysis of BSPM signals, which still implies additional time and cost. Moreover, it requires trained personnel and it involves ionizing radiation in case of CT scan, or it requires compatible equipment in case of MRI. More efficient or alternative solutions to the above factors may help ECGI become a standard tool in ablation procedures.

### Non-invasive Characterization of the AF Organization

When it comes to AF diagnosis and treatment, it is known that patients with similar AF progression (measured in terms of episode duration as paroxysmal, persistent, or permanent) may still respond differently to the same treatment (Kirchhof and Calkins, 2017). This may be due classification based mainly on episode duration may not represent sufficiently well the continuous spectrum of AF progression, ignores the structural component of AF disease, and may overlook subtle differences associated with the corresponding degree of AF substrate complexity. However, taking AF organization into account may form the basis for a more adequate stratification of AF patients (Kirchhof et al., 2012; Lankveld et al., 2014). In this respect, studies on non-invasive mapping AF (Guillem et al., 2009b) already demonstrated different degrees of organization between several patients.

**FIGURE 6:**
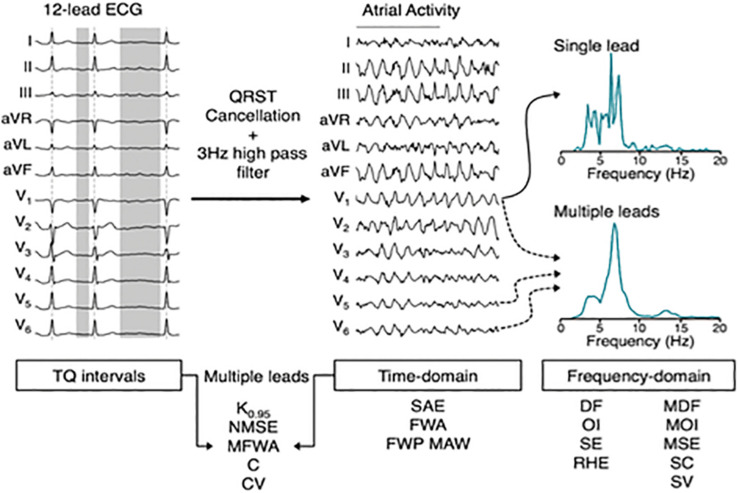
Overview of electrocardiogram (ECG) signal processing and complexity parameter computation. In the time-domain, multi-dimensional parameters derived from multiple leads can be computed on both the extracted atrial activity, as well as on the TQ-segments of the original ECG. In the frequency domain, complexity can be quantified based on spectra computed from a single lead or multiple leads. DF, dominant frequency; OI, organization index; SE, spectral entropy; RHE, relative harmonic energy; MDF/MOI/MSE, multi-dimensional DF/OI/SE; SC, spectral concentration; SV, spectral variability; SAE, sample entropy; FWA, fibrillation wave amplitude; FWP MAW, fibrillation wave power of the main atrial wave; K0.95, C, spatial complexity parameters; NMSE, CV, variability of spatial complexity; MFWA, multi-dimensional FWA. With permission from Zeemering et al. (2018).

In a study based on a goat model of AF, non-invasive measures of AF substrate complexity were computed on invasive recordings, and used to discriminate between short-term and long-term AF (Bonizzi et al., 2015; Figure 6). Results showed that ECG-based measures of AF substrate complexity can discriminate between short-term and long-term AF, and correlate well with standard invasive AF complexity measures (including number of waves, number of breakthroughs, wave size, wave velocity, and atrial fibrillation cycle length). In another study, Zeemering et al. (2018) showed that ECG-based measures of AF substrate complexity in AF patients can improve prediction of successful pulmonary vein isolation and progression to persistent AF compared with common clinical and echocardiographic predictors. A recent study showed that with a suitable pre-processing of the ECG, a more sensitive non-invasive characterization of the AF substrate is possible, which allows to identify short-term atrial activity dynamics significantly associated with AF recurrence in AF patients 4–6 weeks after electrical cardioversion (Bonizzi et al., 2020). These findings from BSPM suggest that the (dis)organization of the atrial propagation patterns, and the corresponding degree of electrophysiological remodeling, do reflect, to a certain extent, on the body surface, that the quality of this information is enough to assess AF progression, and that it can be used for diagnostic purposes.

Localizing AF substrate complexity at the level of the heart is another target for ECGI to stratify AF patients and potentially guide catheter-based ablation. In this respect, non-invasive assessment of AF complexity from BSPM (Meo et al., 2018; Bai et al., 2019) and ECGI (Lim et al., 2017; Rodrigo et al., 2020) has been consistently correlated with ablation outcome. Higher levels of complexity are good predictors of unsuccessful ablation procedures. As for ECGI specifically, already in 2010, Cuculich et al. (2010) demonstrated that ECGI offers a non-invasive way to map epicardial activation patterns of AF in a patient-specific manner. The results highlight the coexistence of a variety of mechanisms and variable complexity among patients and the possibility of being evaluated from ECGI. The distribution of AF patterns has been associated with procedural outcomes and could be used to stratify patients.

### Future of ECGI Validation

ECGI is the first mapping technology that allows a global mapping of both atria simultaneously in a beat-to-beat process. Global mapping has allowed the development of novel AF treatment approaches based on the indication from ECGI maps during AF (Haïssaguerre et al., 2014). Several questions remain open for validation, including what the required resolution is for reliable and accurate reconstruction of the atrial electrical activity, and what the best clinical metrics are for guiding AF ablation.

## Clinical Application: From Clinical Need to Workflow Integration

ECGI may provide a promising opportunity to improve the management of the growing clinical and social burden of AF. A better characterization of the fibrillatory activity offered by the panoramic and non-invasive ECGI mapping of AF simultaneously across the two atria in real time will arguably help patient management for both ablative and non-ablative therapies (Haïssaguerre et al., 2014; Sohns et al., 2017; Rodrigo et al., 2020). Successful application of ECGI in a clinical setting depends on the appropriate clinical goals. Such goals can be divided in two general categories: the first is the opportunity to guide invasive procedures in patients referred for ablation, and the second is to improve patient stratification and treatment selection, possibly leading to a revision of recommendations. The technical requirements for these goals may be different. For example, guiding invasive ablation procedures typically would require the accurate detection and localization of AF drivers such as focal or re-entrant patterns of activation. On the other hand, pre-procedural screening of candidates for successful ablation (or drug therapy) may be based on globally derived metrics of organization and activation rate without the need for precise localization of drivers.

Due to the complex activation nature of AF, with substantial presence of coexisting non-periodic and directionality-varying activation waves in a complex anatomical substrate, clinically meaningful arrhythmia characterization poses a task more challenging in AF than in other cardiac arrhythmias. To facilitate their use and interpretation, the derived ECGI potential maps in the clinic are commonly narrow–band filtered and further processed. This includes transformation of the original signals in the time domain into the phase and frequency domains to extract information about AF mechanisms and complexity (see sections “ECGI in AF: Methodological Considerations” and “ECGI Validation in AF: Outcomes From Mathematical Modeling, Animal Models, and Patients”). For example, activation rate can be extracted from the dominant frequency, and the presence of re-entrant activity can be extracted from phase analysis. Such transformations may allow for a more robust AF characterization approach than others directly based on activation time or wavefront reconstruction.

Table 1 shows the studies identified in the literature where ECGI has been applied to AF (identified through electronic databases including PubMed, Science Direct, IEEE, Scielo, Scopus and Web of Science, from 2000 to 2020; related publications from the Consortium for ECG Imaging (CEI) member were also included). A total of 32 studies were identified (24 original papers, 6 conference papers, 1 conference abstract, and 1 Ph.D. thesis). Around 65% of the studies applied the ECGI technique in AF patients, 10% in both AF patients and AF mathematical models and the remaining only in AF mathematical models. A total of 752 AF patients were reported in the ECGI studies. Around 53% of the studies used CT and 28% an MRI scan to obtain both torso and atria geometries. The remaining studies used realistic 3D models of torso/heart. For the ECGI commercial systems, currently there are three different systems available, ECVUE System (CardioInsight^TM^ Noninvasive 3D Mapping System, Medtronic) with 256 BSPM electrodes and CT imaging modality (FDA and CE Mark), Amycard 01C Noninvasive Epicardial and Endocardial Electrophysiology System (NEEES, EP Solutions SA, Yverdon-les-Bains, Switzerland) with 224 BSPM electrodes and CT/MRI imaging modality (CE Mark), and ACORYS (CORIFY Care SL) with 128 BSPM electrodes and Photogrammetry imaging modality (research only). Epicardial signals were calculated in around 47% of the studies with proprietary commercial solutions (80% with ECVUE-CardioInsight and 20% with Amycard01C-NEEES), representing 82.6% of the studied patients (80.4% from CardioInsight).

**TABLE 1:** Electrocardiographic imaging (ECGI) studies in atrial fibrillation (AF).

References	Applied method of regularization	No. of AF patients	Simulated data	Human data
Wang et al. (2007)	Not specified	1		×
Cuculich et al. (2010)	Not specified	36		×
Haïssaguerre et al. (2013)	Proprietary (ECVUE, CardioInsight, Medtronic Inc.)	2		×
Shah et al. (2013)	Proprietary (ECVUE, CardioInsight, Medtronic Inc.)	52		×
Zellerhoff et al. (2013)	Proprietary (ECVUE, CardioInsight, Medtronic Inc.)	20		×
Haïssaguerre et al. (2014)	Proprietary (ECVUE, CardioInsight, Medtronic Inc.)	103		×
Alday et al. (2016)	Three different orders of the Tikhonov regularization		×	
Dubois et al. (2016)	Tikhonov regularization		×	
Figuera et al. (2016)	Evaluation of 14 regularization techniques		×	
Lim et al. (2016)	Proprietary (ECVUE, CardioInsight, Medtronic Inc.)	90		×
Pedrón-Torrecilla et al. (2016)	Zero-order Tikhonov’s method	4	×	×
Zhou et al. (2016)	Equivalent current density	7		×
Knecht et al. (2017)	Proprietary (ECVUE, CardioInsight, Medtronic Inc.)	118		×
Lim et al. (2017)	Proprietary (ECVUE, CardioInsight, Medtronic Inc.)	105		×
Rodrigo et al. (2017a)	Zero-order Tikhonov’s method		×	
Rodrigo et al. (2017b)	Zero-order Tikhonov’s method		×	
Schuler et al. (2017)	Zero- and second-order Tikhonov regularization		×	
Sohns et al. (2017)	Proprietary [Non-invasive epicardial and endocardial electrophysiology system (NEEES)]	10		×
Suárez-Gutiérrez et al. (2017)	Three different Tikhonov orders		×	
Boyle et al. (2018)	Proprietary (ECVUE, CardioInsight, Medtronic Inc.)	12	×	×
Cochet et al. (2018)	Proprietary (ECVUE, CardioInsight, Medtronic Inc.)	41		×
Rajagopal et al. (2018)	Polynomial neural network	1		×
Rodrigo et al. (2018)	Zero-order Tikhonov’s method	4	×	×
Sohns et al. (2018)	Proprietary [Non-invasive epicardial and endocardial electrophysiology system (NEEES)]	1		×
Metzner et al. (2017)	Proprietary [Non-invasive epicardial and endocardial electrophysiology system (NEEES)]	6		×
Ehrlich et al. (2019)	Proprietary (ECVUE, CardioInsight, Medtronic Inc.)	10		×
Gao et al. (2019)	Proprietary (ECVUE, CardioInsight, Medtronic Inc.)	50		×
Romero (2019)	Zero-order Tikhonov’s method	6	×	×
Molero et al. (2020)	Zero-order Tikhonov’s method	24		×
González-Ascaso et al. (2020)	Zero-order Tikhonov’s method	1		×
Rodrigo et al. (2020)	Zero-order Tikhonov’s method	47		×
Osorio-Jaramillo et al. (2020a)	Proprietary (ECVUE, CardioInsight, Medtronic Inc.)	1		×
Suárez-Gutiérrez et al. (2017)	Three different Tikhonov orders		×	
Boyle et al. (2018)	Proprietary (ECVUE, CardioInsight, Medtronic Inc.)	12	×	×
Cochet et al. (2018)	Proprietary (ECVUE, CardioInsight, Medtronic Inc.)	41		×
Rajagopal et al. (2018)	Polynomial neural network	1		×
Rodrigo et al. (2018)	Zero-order Tikhonov’s method	4	×	×
Sohns et al. (2018)	Proprietary [Non-invasive epicardial and endocardial electrophysiology system (NEEES)]	1		×
Metzner et al. (2017)	Proprietary [Non-invasive epicardial and endocardial electrophysiology system (NEEES)]	6		×
Ehrlich et al. (2019)	Proprietary (ECVUE, CardioInsight, Medtronic Inc.)	10		×
Gao et al. (2019)	Proprietary (ECVUE, CardioInsight, Medtronic Inc.)	50		×
Romero (2019)	Zero-order Tikhonov’s method	6	×	×
Molero et al. (2020)	Zero-order Tikhonov’s method	24		×
González-Ascaso et al. (2020)	Zero-order Tikhonov’s method	1		×
Rodrigo et al. (2020)	Zero-order Tikhonov’s method	47		×
Osorio-Jaramillo et al. (2020a)	Proprietary (ECVUE, CardioInsight, Medtronic Inc.)	1		×

### ECGI for Improved Ablation Guidance

The cumulative density maps of drivers (rotors or focal activity) derived from ECGI have been successfully used for guiding ablation procedures with persistent AF, reaching success rates as high as 85% patients free from AF 12 months after the procedure (Haïssaguerre et al., 2014). As mentioned in section “Validation of ECGI to Guide Ablation Therapy,” these results were confirmed in the AFACART multicenter study that involved centers without prior experience using ECGI mapping systems (Knecht et al., 2017). These findings would contribute to provide a technical basis to approach the main clinical needs in AF: the guidance of ablation procedures, which nowadays presents sub-optimal outcomes for single and multiple procedures (Haïssaguerre et al., 2014; Kirchhof and Calkins, 2017).

An advantage of using MRI to derive the torso-heart geometry is that MRI could be also assisted by deploying Delayed-Enhancement MRI, as another non-invasive modality that can be used to plan and guide ablation procedures in AF patients (Oakes et al., 2009). Accordingly, areas with structural damage, scar progressed fibrosis would appear enhanced in these images and isolation of islands of fibrosis has been proposed as a substrate-based approach for individualizing AF ablation (Akoum et al., 2015). Thus, the combined use of MRI and ECGI may provide the advantage of guiding to possible substrate targets identified by the MRI and to possible AF drivers identified by the ECGI and residing outside of the fibrotic areas (Sohns et al., 2017). The role of the therapeutic MRI-detected atrial substrate modification in patients with persistent AF is currently under evaluation in the ongoing DECAAF II trial (Siebermair et al., 2017).

Recent reports on stereotactic radiotherapy applied to treat ventricular arrhythmias (Cuculich et al., 2017) or atrial fibrillation (Shoji et al., 2019), suggest a non-invasive therapeutic alternative that may expand the possibilities of non-invasive modalities for guiding ablation procedures without catheters.

### ECGI for Improved Patient Selection

**FIGURE 7:**
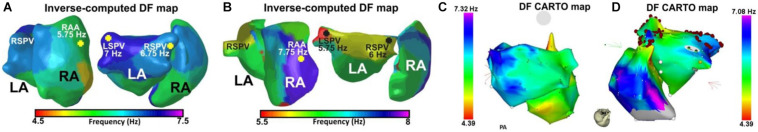
ECGI-based **(A,B)** and intracardiac-based **(C,D)** dominant frequency maps of patients with a left-to-right dominant frequency gradient **(A,C)** or a right-to-left dominant frequency gradient **(B,D)**. Modified from Pedrón-Torrecilla et al. (2016).

Although ablation procedures are generally safe, they are not totally free of risks, require substantial resources, and are not indicated for all AF patients (Cappato et al., 2010; Khaykin and Shamiss, 2012). An ECGI-based panoramic mapping of AF simultaneously across both atria may enable pre-procedural screening and planning of ablation procedures to possibly reduce these resources and risks. As a possible paradigm for such an approach, the study by Atienza et al. (2009) found that AF showing no inter-atrial gradients of dominant frequencies did not terminate by ablation and therefore, the ECGI is envisioned as being able to provide a quantitative index to better screen patients for ablation or other therapeutic procedures. This frequency-based screening approach seems viable as the dominant frequency mapping during AF by the BSPM, a first step of the ECGI procedure, has been validated by comparing the dominant frequency atrial maps based on EGGI with maps based on intracardiac recordings (Figure 7; Haïssaguerre et al., 2014; Pedrón-Torrecilla et al., 2016). A direct support for the screening possibility was provided by a study by Meo et al. (2018) showing that BSPM contains sufficient information on the complexity of spatiotemporal patterns of atrial electrical activation to predict which patients may benefit from ablation therapy, and may thus serve to select patients suitable for this invasive therapy. The increasing complexity of non-invasive AF patterns with longer AF duration was associated with a decline in acute termination rates and decreased long-term efficacy, underscoring the importance of early AF treatment (Kirchhof et al., 2020). In the same direction, a study by Rodrigo et al. (2020) showed that complexity of electrical patterns projected reliably on the atrial surface can be derived from ECGI measurements and thus may serve in stratifying patients and recommend for ablation preferentially those with less complex electrical substrates.

Overall, the possibility of characterizing the atrial activation patterns non-invasively by ECGI is an incentive for further development of this technology to predict the outcome and evaluate the effect of both ablative and non-ablative AF therapies by directly monitoring the electrical activity in the atria. One challenging development also needs to address the derivation of ECGI metrics employed for screening and guiding ablation procedures of AF patients presented to the ECGI evaluation in sinus rhythm. These new potential applications of ECGI have a much wider potential use in clinical practice than simple ablation guidance and may become the preferred approach for patients’ clinical management. Furthermore, the STRATIFY-AF study is currently analyzing the role of non-invasive risk ECGI-based stratification of ambulatory AF patients and could serve as a basis for establishing novel management approaches (Atienza et al., 2021).

**FIGURE 8:**
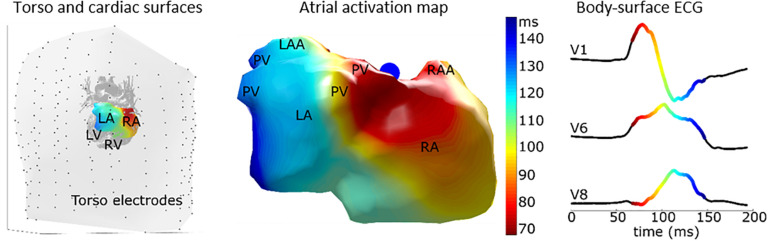
Epicardial activation pattern of an individual without a history of AF, highlighting the level of detail with which the activation pattern can be studied on the atria to better understand normal patterns and diseased patterns that may reflect AF substrate. The left panel shows the torso electrodes (black dots) and torso (gray surface) with cardiac structures inside. The middle panel shows the atrial epicardial activation sequence as mapped with Electrocardiographic imaging (ECGI). The right panel shows corresponding body-surface P-waves of leads V1, V6, and V8. LA, left atrium; RA, right atrium; LV, left ventricle; RV, right ventricle; PV, pulmonary vein; LAA, left atrial appendage; RAA, right atrial appendage. Figure modified from Lankveld (2016).

Even in the absence of AF, studying atrial activation patterns from BSPM recordings has been shown to be informative on the substrate for AF (Schill et al., 2020). ECGI has previously been applied to non-invasively study the P wave (Figure 8), and extension of such analysis to study the substrate for AF in sinus rhythm may help predict the effect of therapy.

### Implementing ECGI in a Clinical Workflow

Despite intensive research and development efforts, the clinical adoption of ECGI for AF ablation is currently limited. Several factors may contribute to such limited use. First, as discussed above, accurate and validated maps of the atrial source signals are difficult to reconstruct in ECGI and until reliable mapping is demonstrated clinicians will be hesitant to adopt the approach. Second, mechanisms of AF maintenance and termination by ablation are not fully understood, which may also reduce the endorsement of any AF mapping system by the clinicians. In comparison, the ECGI method has been gaining an increasing clinical credibility for VT ablation following demonstration of success cases (Cuculich et al., 2017). In AF, Haïssaguerre et al. demonstrated the clinical potential of ECGI in AF, where over hundred persistent AF patients were enrolled (Haïssaguerre et al., 2014). Further independent studies on complex and less understood AF, would be of importance to increase worldwide clinic adoption of ECGI for AF.

In addition, practical workflow hurdles can also possibly explain the limited usage of ECGI in AF. The ECGI approach is logistically challenging as it depends on a combination of imaging, mapping and ablation procedures requiring seamless coordinated efforts by various clinical and technical support teams. The efforts include a pre-procedure selection and testing of the multi-electrode body surface vest to fit the patient habitus and then keeping the vest tight and in position during the CT/MRI scan and the following AF mapping and ablation procedure. The time between CT/MRI and any EP procedure may last >4 h to allow for processing of the image data, generation of the torso-heart geometry, and preprocedural EP lab preparations or wait-time. These lengthy protocols may introduce another limitation: although during the procedures multiple movies and maps can be readily reconstructed about 30 min after each electrical recordings’ episode, the quality of the maps may be diminished with time, because over time the electrodes in the vest can become more prone for displacement, which has been found to affect more than 20% of the recordings.

The successful clinical implementation of ECGI for AF management requires defining metrics of clinical success, such as freedom from AF and improved quality of life, vs. economic metrics of long-term financial investment and reduction in AF therapy costs, as well as adjustments of workflow and infrastructure. Important components for the effectiveness of the ECGI approach in successful AF management are its hardware fidelity and the specialized training of operators and physicians, both critical for a standardized high-quality operation of the approach. Recent studies highlighted the limitations in the optimization procedures (Bear et al., 2015) and accurate determination of spatiotemporal patterns of epicardial potentials (Duchateau et al., 2019) based on ventricular recordings. It has been suggested that those limitations could be possibly overcome by improved hardware, which will require increased industry effort, as well as more careful registration of body-heart and electrode (vest) geometry and properties, which will require a better operation of the ECGI system and procedures by staff at the clinical settings (Rudy, 2015, 2019).

The adoption of ECGI in the clinical practice is predicted to continue to be challenging with a likely requirement in modifications of traditional workflow and infrastructures. In either the pre- or intra-EP procedure setting, the CT/MRI imaging modalities used in ECGI will need to be better integrated with the EP modalities for recording and analysis, as well as ablation, to optimize ECGI usage. That integration is predicted to require an infrastructure retrofitting to clinics, which is seen as a major financial impediment for adoption of the new technology (Greenhalgh et al., 2017a,b). As an alternative, other solutions focus on possibly eliminating the CT/MRI modality from the ECGI workflow (Rodrigo et al., 2020) to facilitate the torso-heart geometrical registration procedure.

## Discussion

Atrial fibrillation substrate mapping is one of the main challenges of cardiac electrophysiology. During the last two decades endocardial mapping has failed to provide a stable methodology to identify AF ablation target regions. ECGI is nowadays the only technology that can provide mapping of both atria simultaneously and has demonstrated limited but promising clinical efficacy to guide ablation procedures. However, performing ECGI adequately in the clinical practice is a complex process. For any application, ECGI is challenging due to its inherent instability (ill-posedness, which can be partially tackled by regularization methods) and required clinical investments (application time, need for specialized operators, and dedicated imaging). On top of these general ECGI challenges, the signal processing of atrial signals (generally having a lower amplitude on the body-surface recording) and disorganized signals (during AF) is more challenging than reconstructing ventricular or “regular” signals. Balancing these trade-offs, most applications of ECGI for atrial signals have reverted to using approaches that are less sensitive to noise (typically reconstructing epicardial potentials with Tikhonov regularization) but may also deliver a relatively low resolution. Further post processing of such signals has proven to be essential to extract relevant information, and may include translation from the reconstructed time-domain signals to the frequency domain. For example, it was shown that reconstructing electrograms during AF may be challenging, but that phase and spectral analysis of such signals can still provide relevant information on activation sequences, activation rates and regions with dominant rotational frequencies.

As with any AF treatment innovation, it remains an ongoing challenge to validate that such information is relevant in guiding AF management. Additionally, ECGI *for* AF does not necessarily mean ECGI *in* AF, and studying atrial activation patterns in sinus rhythm may already be informative on the underlying (structural) substrate.

Classifying AF in subtypes (which may have relevance for therapy) has been performed repeatedly and on different signal sources (12-lead ECG, BSPM, ECGI, invasive recordings or imaging data). ECGI may provide an attractive alternative to go beyond the “persistent” vs. “paroxysmal” classification that is purely based on AF duration. Non-invasive ECGI metrics may yield a more accurate representation of the mechanisms and substrate for AF, and may thus be better suited for guiding AF management. Even if ECGI classification metrics significantly overlap and thus may not be of significance for an individual patient, they may still teach us more about the mechanisms of AF. Improvements would allow us to select patients for therapy (e.g., derive and evaluate metrics that predict ablation outcome/success). Next steps include localizing abnormalities which can guide invasive ablative therapy to specific myocardial regions, for improved outcome.

Clinical application of ECGI as a standard tool for guiding AF treatment would require improvements in the technology to streamline workflow and an extensive validation of patients’ stratification and ablation guidance efficacy. The combination of electrical mapping through ECGI with other auxiliary clinical techniques could potentially advance understanding of arrhythmia mechanisms and treatment options. Such advances are particularly important for AF, which is the most common arrhythmia with inter-patients heterogeneity with both electrical and structural remodeling. Whereas, ECGI may provide novel insights into the electrical activity, structural imaging (CT, MRI, ultrasound) can provide complementary information which may be of particular importance in multi-factorial diseases such as AF. At an individual patient level, combining pre-procedural ECGI with pre-procedural structural imaging and intra-procedural mapping may lead to higher diagnostic and therapeutic value.

**FIGURE 9:**
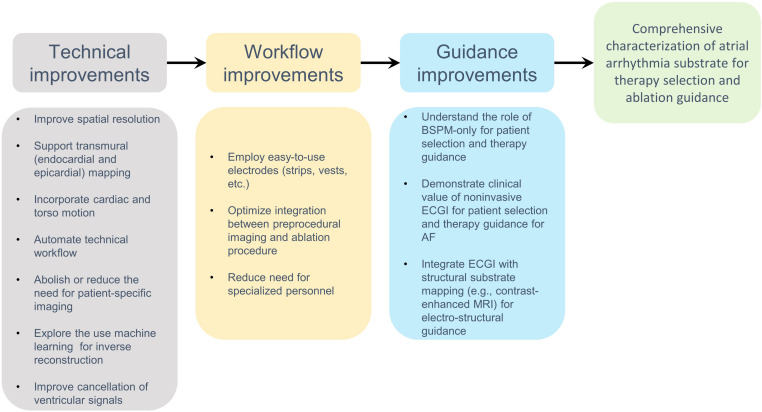
Roadmap with future directions of Electrocardiographic imaging (ECGI) in atrial fibrillation (AF).

Integration and adoption of ECGI in clinical setting will be a reality only once it has been shown to add substantial benefits. Improving the integration of ECGI in a clinical workflow will also depend on its need for specialized personnel and pre-procedural imaging. Simultaneously, if ECGI could be used to speed up AF ablation procedures, overall workload reduction could be achieved. Combining workflow improvement, decreased ablation procedural times with improved patient outcome would then support reimbursement and implementation in the daily clinical management of AF. Until those technological challenges are overcome, ECGI for AF is a powerful research tool and provides clear benefits to better understand AF mechanisms. Figure 9 presents a roadmap summarizing some of the possible future directions for the ECGI.

## Conclusion

The development of ECGI for better understanding AF mechanisms and potentially guiding therapy has a long and rich history. Its potential immediate value may lie particularly within its ability to help understanding AF mechanisms and the effects of treatment. In the future, ECGI may be better integrated with cardiac structure-function imaging modalities and other approaches. The combination of those approaches holds the promise that ECGI may be developed further to deliver a much-needed non-invasive electrical mapping approach that is key to guide AF treatment.

## Author Contributions

JS, FS, MR, OB, RMo, MG, and PB conceived of the presented idea, contributed to the design, and implementation of the research. JS, MC, and PB involved in supervising the work and contributed to the final manuscript. JS, RMo, FS, JK, MR, JR-Á, OB, AC, BZ, FV, RM, FA, MG, MC, and PB contributed to the interpretation and revision of the results. All authors worked out with the technical details, wrote the manuscript, and approved the submitted version.

## Conflict of Interest

MC is part-time employed by Philips Research (Eindhoven, Netherlands). AC, MG, and FA have equity of Corify Care (Madrid, Spain). AC is part-time employed by Corify Care (Madrid, Spain). FA served on the advisory board of Medtronic and Microport. OB was co-founder and Scientific Officer of Rhythm Solutions, Inc., consultant to Acutus Medical and is a co-founder of Cor-Dx LLC. The remaining authors declare that the research was conducted in the absence of any commercial or financial relationships that could be construed as a potential conflict of interest.

## Footnotes

1 ecg-imaging.org
