# Supplementary material for: Electrocardiographic Imaging for Atrial Fibrillation: A Perspective From Computer Models and Animal Experiments to Clinical Value
Source: Front Physiol. 2021 Apr 30;12:653013. doi: 10.3389/fphys.2021.653013 (PMC8120164; doi:10.3389/fphys.2021.653013)
Supplement: Supplementary file 1 [file Presentation_1.pdf]

Supplementary Material

1. Physics description of ECGI

Electrocardiographic imaging (ECGI) is a medical tool that allows estimating electrical activity on the heart surface based on non-invasive signals obtained from 3D geometries of the torso (Rudy, 2013). This technology has been widely used in the study of several heart diseases, as it allows analyzing the electrical activity of the heart in a way comparable to invasive techniques (catheterization), but with greater coverage and offering improved versatility, as it allows the study and planning of surgical therapy before the procedure (Rudy, 2013).

The physics of ECGI is governed by the Maxwell electromagnetic wave equation, which allows us to infer that the average value of the electric potential around a point P is equal to the value of the potential at t point P, this is, the electromagnetic wave equation is given by

$$\nabla^2 \phi = 0 \tag{1}$$

where ∇^2 represents the Laplacian operator and ϕ (mV/m) is the electric field generated by the excitation of the heart, and which is propagated to the surface of the body through a volume with passive electrical conduction (Rudy and Burnes, 1999; Rudy, 2013). In the ECGI method, potentials in the heart ϕ_C (mV) are calculated using the Laplace equation within the torso volume conductor, using both the electrical potentials from the torso, $\phi_T(mV)$, and the geometric relationship between the 3D surfaces of the heart and torso (A_{TC}) (Rudy and Burnes, 1999; Rudy, 2013). The first step in this procedure is the discretization of the 3D surfaces of the heart and torso into triangular elements allowing the relationship of ϕ_T and $\phi_C$to be calculated through the transfer matrix A_{TC} as follows,

$$\phi_T = A_{TC}\, \phi_C \tag{2}$$

Discretization is achieved through the Finite Difference, Finite Element or Boundary Element Methods (FDM, FEM and BEM, respectively), resulting in a linear relationship matrix (this transfer matrix A_{TC}), which contains the geometric information and electrophysiological properties of the volume conductor that relates the two surfaces (atrium and torso) (Rudy and Burnes, 1999; Rudy, 2013).The direct solution of Eq. (2) for calculating ϕ_T is a well-posed mathematical problem in the sense of Hadamard (that is, the solution exists, is unique and stable) (Rudy and Burnes, 1999; Kabanikhin, 2008; Rudy, 2013). This means that given a geometric relationship between the heart and the torso and a set of potentials on the epicardium, the potentials on the surface of the torso can be calculated with high accuracy (Rudy and Burnes, 1999; Rudy, 2013). This does not hold for the inverse problem, because typically $A_{TC}$is not a rectangular matrix, or if it is square, it is ill-conditioned or degenerate. Moreover, we must observe that assuming A_{TC} as a constant matrix reality is simplified, since e.g. there is an interaction between A_{TC} and ϕ_C(movement and contraction of the heart) and $A_{TC}$is dynamic due to respiration. A further complicating factor is that we can only estimate A_{TC}.

The objective of ECGI is to obtain the epicardial potentials (ϕ_C) from the potentials measured on the torso (ϕ_T) (Bertero and Boccacci, 1998). As argued, this inverse problem is ill-posed, leading

to stability issues, i.e. small perturbations either in the measurement of the torso signals (i.e. noise and / or position of the electrodes), or in the 3D geometries of the torso and heart, can result in large errors in the calculation of ϕ_C (Colli-Franzone et al., 1985; Rudy and Burnes, 1999; Kabanikhin, 2008; Pullan et al., 2011; Rudy, 2013). Moreover, the solution for ϕ_C in (Eq. 2) may not exist for all elements of ϕ_T, or there might be many solutions.

Regularization methods are used to force a unique and smooth solution, with Tikhonov regularization (Tikhonov and Arsenin, 1977) being one of the main set of methods for this aim, as it performs as well as other more complex methods under realistic fibrillatory conditions (Figuera et al., 2016). This method imposes limits in ϕ_C delimiting the amplitudes or derivatives of the epicardial potentials in space, time, or both, to be within the electrophysiological and electric field limits of the heart (Rudy, 2013). Regularization is an approach to find an approximation of ϕ_C that depends continuously on the noisy measurement data ϕ_T, but the chosen regularization should bring the estimate of ϕ_C as close to its true value as the noise levels (on both ϕ_C and A_{TC}) permit. Prior information can be added to the regularization, such as further limiting the solution space (Kabanikhin, 2008). Through regularization, it is possible to find a solution to the inverse problem of electrocardiography.

Figure 1 in the main text illustrates the standard steps needed for the implementation of ECGI systems. Values in the A_{TC} correspond to estimated electrophysiological properties of nodes in a mesh describing the heart and body geometries as well as tissues and organs between the heart and body surface. The mesh is usually generated from medical images (either CT or MRI). Through this approach, the regularized inverse of the matrix that relates the geometries of the torso and heart (A_{TC}) in Eq. (2) can be calculated. After calculating the regularized inverse matrix, the estimation of the potentials in the atrium ϕ_C can be estimated through the product of this matrix with the potentials acquired from the torso ϕ_T using body surface potential mapping (BSPM) systems.

Calculation of the elements of the transfer matrix

To calculate the elements of transfer matrix A_{TC}, it is necessary to consider first the unknown potential $\phi(\vec{x})$(mV) given by the solution to the following contour integral equation (de Munck 1992):

$$\frac{\Omega(S;\vec{x})}{2\pi}\phi(\vec{x}) = 2\phi^{\infty}(\vec{x}) - \frac{1}{2\pi}\oiint_{S\backslash\exists(\vec{x})}\phi(\vec{x}')\nabla'\frac{1}{|\vec{x}'-\vec{x}|}\cdot\ d\vec{S}' \tag{3}$$

In this equation, we consider the calculation of the potential ϕ $(\vec{x})$ caused by a current source with constant and isotropic conductivity, and within an area bounded by surface S. Here, $\phi^{\infty}(\vec{x})$ is the potential generated in a medium of infinite extension and $\Omega(S;\vec{x})$ is the solid angle of surface S, as seen from point $\vec{x}$. If $\vec{x}$ is positioned on S, the solid angle equals 2π. We denote as $S\backslash\exists(\vec{x})$ to indicate that the integral is assumed on surface S with which an environment of $\vec{x}$ is excluded. Then, it is assumed the limit to $\hat{E}(\vec{x})\rightarrow\{\vec{x}\}$.

Solution of Eq. (3) can be approximated by choosing a set of functions $\{h_n(\vec{x}_m)\}^{N}_{n=1}$ and a set of discretized points $\{\vec{x}_m\}^{N}_{n=1}$ so that:

$$h_n(\vec{x}_m) = \delta_{nm} \quad (4)$$

where δ_{nm}represents a Delta function as the result of evaluating $h_n(\vec{x}\ \ )$in the discretized point $\vec{x}_m$. The unknown potential field $\phi(\vec{x})$ is expanded in terms of $h_n(\vec{x}\ \ )$, this is,

$$\phi(\vec{x}) = \sum_{n=1}^{N} \quad \phi_n h_n(\vec{x}) \quad (5)$$

where ϕ_n are the coefficients of said expansion. With the above definitions, Eq. (1) can be approximated to a set of linear equations with *N* different values of

$$\sum_{n=1}^{N} \quad B_{mn}\phi_n = 2\phi^{\infty}(\vec{x}_m) \quad (6)$$

where the matrix elements are calculated by

$$B_{mn} = \frac{1}{2\pi}\left(\oiint_{S\backslash\exists(\vec{x}_m)} h_n(\vec{x}')\nabla' \frac{1}{|\vec{x}'-\vec{x}_m|} \cdot \ d\vec{S}' \ + \ \delta_{nm}\Omega(S;\vec{x}_m)\ \right) \quad (7)$$

The simplest choice for the functions is obtained from the triangulation of *S* and by making $h_n(\vec{x})$ equal to 1 at the nth triangle and zero at other triangles. The discretized points $\vec{x}_m$ are given by the centers of the triangles. In this approach, the potential is described as a constant function defined by parts, therefore, many triangles are necessary to accurately represent the potential. In the case where the approximation is made by linear interpolation of the potential, surface *S* is also subdivided into small triangles, but unlike the approximation by a constant potential, the discretization points are given by the vertices of the mesh. The interpolation functions in this approximation are given by:

$$h_n(\vec{x}) = \frac{det(\vec{x}_k, \vec{x}_l, \vec{x})}{det(\vec{x}_k, \vec{x}_l, \vec{x}_n)}, if \ \ \vec{x} \ \in \ \Delta_{kln}, otherwise \ h_n(\vec{x}) = 0 \quad (8)$$

where $\vec{x}_k, \vec{x}_l, \vec{x}_n$are the vertices of triangle Δ_{kln}, and $h_n(\vec{x})$ is a linear function defined by parts of $\vec{x}$ and satisfying the condition in Eq. (2). To calculate the matrix elements, the integration area is partitioned into triangles adjacent to $\vec{x}$. In each triangle, a normal $\vec{n}$ is independent of $\vec{x}$. Moreover, the diagonal elements are given separately, in such a way that we can assume $n \neq m$. Then, it can be readily shown that

$$B_{mn} = \frac{-1}{2\pi}(\sum_{\Delta_{kln}} \quad \frac{detdet\ (\vec{x}_k-\vec{x}_m, \vec{x}_l-\vec{x}_m, \vec{x}_n-\vec{x}_m)}{A_{kln}} \cdot \iint_{\Delta_{kln}} \frac{h_n(\vec{x}')}{|\vec{x}'-\vec{x}_m|^3}\ dS' \quad (9)$$

where the sum is on all adjacent triangles to $\vec{x}_n$and where A_{kln} is the area of triangle Δ_{kln}. To find the diagonal elements, and knowing that $\sum_n \quad h_n(\vec{x}\ ')$=1, the vector (1, 1, …, 1) is the eigenvector with *B*, corresponding to eigenvalue zero, as follows:

$$\sum_{n=1}^{N} \quad B_{mn} = \frac{1}{2\pi}\left(\oiint_{S\backslash\exists(\vec{x}_m)} \nabla' \frac{1}{|\vec{x}'-\vec{x}_m|} \cdot \ d\vec{S}' + \Omega(\vec{x}_m)\right) = 0 \quad (10)$$

Through this property of *B*, we have that its diagonal elements can be expressed in terms of the other remaining matrix elements, such that:

$$B_{mm} = -\sum_{n\neq m}^{N} \quad B_{mn} \tag{11}$$

Therefore, for the calculation of the linear relation matrix (A_{TC}) that relates the geometries of the torso and the atria, it is necessary to triangulate the surfaces to generate meshes defined by vertices and faces, where each vertex is a point of discretization. This triangulation is often derived from segmentation of medical images of the torso.

Non-proprietary algorithms

From the studies that used non-proprietary algorithms for inverse solution, mostly applied the zeroth-order Tikhonov regularization method (n=13; 40%). Bayes maximum-a-posteriori regularization method outperformed the most common regularization techniques but this technique requires prior information about the epicardial potentials, which usually is not available in the majority of cases in clinics. Tikhonov-based methods performed as well as more complex techniques in realistic fibrillatory conditions.

Tikhonov regularization method

The Tikhonov regularized solution is obtained by minimizing an appropriate objective function (Pullan et al., 2011)

$$\phi_C \ = \ min \,[\| \ A_{TC} \ - \ \phi_T|| \ \ {}_2{}^2 + \lambda \quad \| \ R\phi_C \ \|^2{}_2 \ \ ] \tag{13}$$

where R is the $N \times N$ Tikhonov matrix and $\| \ \cdot \|_p$ is the p-norm. Matrix R helps to restrict (hence to regularize) the inverse solution. The first term $\| \ A_{TC} \ - \ \phi_T \ \ ||_2{}^2$ represents the L2 squared error, while the second term restricts, in the spatial domain, the energy of the solution according to the choice of the particular Tikhonov matrix R. Here λ is called the regularization parameter, and it determines to what extent the final inverse solution will depend on R. A higher λ leads to a smoother solution (i.e. reduces more noise), but it can remove localised activation patterns (an over-smoothed solution) (MacLeod and Buist, 2010). There are three Tikhonov matrices (Pullan et al., 2011) that are commonly used in inverse electrocardiography. Zero-order Tikhonov regularization sets $R = I$, the identity matrix, which effectively limits the total magnitude of the solution. First-order Tikhonov regularization sets $R = G$, a discrete approximation of the surface gradient operator that limits the slope of the solution. Finally, Second-order sets $R = L$, a discrete approximation of the Laplacian surface operator, to restrict the rate of change in the slope. The use of zero-order Tikhonov method, even with a constant regularization parameter, is justified by the fact that it is a good alternative to solve the inverse problem of electrocardiography during AF. Comparisons made between different regularization methods used to calculate atrial potentials during AF suggest that the zero-order Tikhonov regularization method might be insensitive to moderate changes in regularization parameters. In addition, results for different values of signal-to-noise ratio (SNR) showed that no algorithm was significantly more robust regarding to changes in noise level (Figuera et al., 2016).

With Tikhonov regularization, we can write a closed solution for Eq. (13) as

$$\phi_C = \ (\, A_{TC}{}^T \, A_{TC} \ + \ \lambda \ \ R^T R)^{-1} \, A_{TC}{}^T \phi_T \tag{14}$$

Hence, in the unregulated case (λ=0), the Moore-Penrose pseudoinverse is obtained. Replacing Eq. (14) in Eq. (3), the solution for zero-order Tikhonov regularization (Pullan et al., 2011) is

$$\phi_C = (A_{TC}{}^T A_{TC} + \lambda \ I)^{-1} A_{TC}{}^T \ \phi_T \tag{15}$$

with $A_{TC}{}^T$ denoting the transpose matrix of A_{TC}.

Other regularization methods

Other regularization methods that have been implemented for smoothing the inverse solution include Generalized Minimal Residual (GMRes), singular value decomposition (SVD), total variation (TV), Bayesian Maximum a Posteriori Estimation (Bayes) and MUltiple SIgnal Classification (MUSIC/Greensite) algorithms (Figuera et al., 2016; Ghosh and Rudy, 2009; Pereyra, 2017; Onal and Serinagaoglu, 2009; Ramanathan et al., 2003).

The GMRes method is an iterative numerical method that does not require to impose constraints on the solution (Ramanathan et al., 2003, Calvetti et al., 2000). In this method, the inverse of matrix A_{TC} is approximated by its projection $p_n(A_{TC})$ onto a Krylov subspace *K(n)*, which comprises the set of all linear combinations of the vectors in ϕ_T(Ramanathan et al., 2003). The GMRes method requires A_{TC} to be square and ϕ_T to be normalised (Calvetti et al., 2000). As such, the solution to be minimised becomes (cfr. Eq. 13)

$$\left|\left|A_{TC}\,\phi_T - \phi_C\right|\right| = min\left|\left|H_m\widehat{\phi_T} - \widehat{\phi_C}e_1\right|\right| \tag{16}$$

With $H_m$denoting an upper Hessenberg matrix, $e_1$the first axis vector of an Arnoldi decomposition of $A_{TC}$and $\hat{x}$the approximate solutions to the torso and cardiac potentials.

The most popular SVD algorithms are truncated (TSVD) and damped (DSVD) SVD. In TSVD, the matrix $A_{TC}$is truncated such that all its singular value components that represent noise are removed. As components representing noise usually have small singular values, the truncation is implemented by maintaining a set of *k* components with the highest singular values (Hansen, 2010). The value of *k* needs to be set a priori to obtain a solution, and the function to be minimised reads:

$$\phi_C = min\,[\| A_{TC,k} - \phi_T||_{\ 2}\ ] \tag{17}$$

DSVD is a less ‘brute force’ application compared to TSVD, as it allows a filtering of singular value components rather than forcing to make an inclusion/exclusion decision on the components to include in the final solution (Figuera et al., 2016).

TV applies an L_1-norm penalization on the inverse solution, rather than an L_2-norm in Tikhonov regularization. The function to minimise therefore becomes (Figuera et al., 2016):

$$\phi_C = min\,[\| A_{TC} - \phi_T||_{\ 2} + \lambda \ \ \| R\phi_C \|_{\ 1}\ ] \tag{18}$$

The Bayesian approach is based on a priori knowledge of the spatial covariance matrix and mean of the epicardial potentials. Setting this mean to zero gives (Figuera et al., 2016):

$$\phi_C = (C_c A_{TC}{}^T)(A_{TC} C_c A_{TC}{}^T + C_n)^{-1} \phi_T \tag{19}$$

With C_c and C_n the covariance matrices of the epicardial potentials and noise. This approach only accounts for spatial correlation of the potentials. A temporal correlation can be included based on the isotropy assumption described by Greensite (2003). Here, the covariance matrix C_c is extended to $C_c = C_t \otimes C_x$, with $C_t$the temporal covariance matrix and C_x the spatial covariance matrix (Figuera et al., 2016).

References

Bertero, M., and Boccacci, P. (1998). Introduction to Inverse Problems in Imaging. CRC Press.

Calvetti, D., Lewis, B., and Reichel, L. (2000). Restoration of images with spatially variant blur by the GMRES method in Proceedings of the SPIE, Advanced Signal Processing Algorithms, Architectures, and Implementations X 4116, 364-74.

Colli-Franzone, P., Guerri, L., Tentoni, S., Viganotti, C., Baruffi, S., Spaggiari, S., and Taccardi, B. (1985). A mathematical procedure for solving the inverse potential problem of electrocardiography. Analysis of the time-space accuracy from in vitro experimental data. Math Biosci 77(1-2): 353-96.

Figuera, C., Suárez-Gutiérrez, V., Hernández-Romero, I., Rodrigo, M., Liberos, A., Atienza, F., Guillem, M.S., Barquero-Pérez, Ó., Climent, A.M., and Alonso-Atienza, F. (2016). Regularisation techniques for ecg imaging during atrial fibrillation: a computational study. Front Physiol 14(7), 466.

Ghosh, S., and Rhee, E.K., and Avari, J.N., and Woodard, P.K., and Rudy, Y. (2008). Circulation 118(9):907-15.

Greensite, F. (2003). The temporal prior in bioelectromagnetic source imaging problems. IEEE Trans Biom Eng 50(10), 1152-59.

Hansen, P. C. (2010). Discrete inverse problems: insight and algorithms. Philadelphia : Society for Industrial and Applied Mathematics (SIAM).

Kabanikhin, S. I. (2008). Definitions and examples of inverse and ill-posed problems. J Inverse Ill Posed Probl 16(4), 317-357.

MacLeod, R., and Buist, M. (2010). The forward problem of electrocardiography in Comprehensive electrocardiology, eds. P.W. Macfarlane, A.v. Oosterom, O. Pahlm, P. Kligfield, M. Janse, J. Camm (London, Springer), 247-298.

Munck, J.C. (1992). A linear discretization of the volume conductor boundary integral equation using analytically integral elements. IEEE Trans Biomed Eng 39(9), 986-9902.

Pereyra, M. (2017). Maximum-a-posteriori estimation with Bayesian confidence regions. SIAM Journal on Imaging Sciences 10(1), 285-302.

Pullan, A.J., Cheng, L.K., Nash, M.P., Ghodrati, A., MacLeod, R., and Brooks, D.H. (2011). The Inverse Problem of Electrocardiography in Comprehensive Electrocardiology, eds. P.W. MacFarlane, A.v. Oosterom, O. Pahlm, P. Kligfield, M. Janse, J. Camm (London, Springer), 300-336.

Ramanathan, C., Jia, P., Ghanem, R., Calvetti, D., and Rudy, Y. (2003). Noninvasive electrocardiographic imaging (ECGI): application of the generalized minimal residual (GMRes) method. Ann Biomed Eng. Sep 31(8), 981-94.

Rudy, Y., and Burnes, J. (1999). Noninvasive Electrocardiographic Imaging. Ann Noninvasive Electrocardiol 4(3): 340-59.

Rudy, Y. (2013). Noninvasive electrocardiographic imaging (ecgi) of arrhythmogenic substrates in humans. Circulation Research 112(5):863-74.

Tikhonov, A.N., and Arsenin, V. (1977). Solutions of ill-posed problems. New York: John Wiley & Sons.
